# Sensitivity to oxazolone induced dermatitis is transferable with gut microbiota in mice

**DOI:** 10.1038/srep44385

**Published:** 2017-03-14

**Authors:** Line Fisker Zachariassen, Lukasz Krych, Kåre Engkilde, Dennis Sandris Nielsen, Witold Kot, Camilla Hartmann Friis Hansen, Axel Kornerup Hansen

**Affiliations:** 1 Department of Veterinary and Animal Sciences, Faculty of Health and Medical Sciences, University of Copenhagen, Denmark; 2 Department of Food Science, Faculty of Science, University of Copenhagen, Denmark; 3 The Bartholin Institute, Rigshospitalet, Copenhagen, Denmark; 4 Department of Environmental Science, Faculty of Science and Technology, Aarhus University, Denmark

## Abstract

Atopic Dermatitis (AD) has been associated with gut microbiota (GM) dysbiosis in humans, indicating a causative role of GM in AD etiology. Furthermore, the GM strongly correlates to essential disease parameters in the well-known oxazolone-induced mouse model of AD. Here, we demonstrate that it is possible to transfer both a high-responding and a low-responding AD phenotype with GM from conventional mice to germ-free mice. The mice inoculated with the high-responding GM had significantly higher clinical score, increased ear thickness, and increased levels of IL-1β, TNFα, IL-4, IL-5, and IL-6 compared to the mice inoculated with the low-responding GM. The inter-individual variation was in general not affected by this increase in effect size. Germ-free mice induced with AD revealed a high disease response as well as high inter-individual variation indicating protective properties of certain microbial taxa in this model. This study underlines that the GM has a strong impact on AD in mouse models, and that the power of studies may be increased by the application of mice inoculated with a specific GM from high responders to increase the effect size.

Atopic dermatitis (AD) is a common skin disease characterized by inflammatory, noncontagious, pruritic eczema^1^. AD is driven by contact with environmental allergens, which often in their form of haptens become immunogenic when reacting with host proteins. Pathogenesis includes skin barrier disruption, genetic predisposition^2^, elevated IgE levels and immune dysregulation characterized by initial T helper cell type 2 (Th2) and T helper cell type 1 (Th1) in the chronic phase^3^,^4^,^5^.

Stress, infections, and exposure to food- and aeroallergens all contribute to the disease^6^,^7^. The hygiene hypothesis proposed by Strachan in 1989, postulates that a decreased exposure to bacteria early in life due to increased hygiene in the Western industrialized countries results in impaired immune system activation and regulation leading to higher prevalence of allergies and atopic diseases^8^,^9^. This could explain the higher prevalence of AD in Western industrialized countries being more sanitized than developing countries^10^,^11^,^12^.

Human studies have revealed gut microbiota (GM) compositional changes in AD patients compared to healthy controls. In general, patients with AD have a less diverse GM, with a lower abundance of *Bifidobacterium* spp. while being more frequently colonized with *Staphylococcus aureus*, certain clostridia, and *Escherichia coli*^13^,^14^,^15^. The amount of *E. coli* in the GM further correlates to serum IgE concentrations^16^. *Bifidobacterium* spp. has an alleviating effect, showing a positive effect of probiotics in both prevention and treatment of AD^17^,^18^,^19^. GM composition changes are demonstrated early in life prior to any clinical manifestations, indicating that dysbiosis is causative rather than an effect of AD^20^,^21^.

A well-known model for AD-like symptoms is the oxazolone-induced mouse, in which multiple epicutaneous administration of oxazolone, a potent hapten, induces a chronic Th2 hypersensitivity reaction resembling the features of initial human AD. Clinically, this model is characterized by a thickened ear/oedema, haemorrhagia, excoriations, and lichenefication^22^,^23^. In this model, the GM has shown to strongly correlate with ear tissue levels of IFN-γ, TNF-α, and IL-10^24^.

Previously, a causal relationship between GM and obesity^25^, colitis^26^ and enteric infections^27^ has been demonstrated by fecal transfer of phenotypes to germ-free mice. However, it has never been demonstrated that sensitivity to chronic oxazolone inflammation, resembling AD, can be transferred with the GM between mice. This is of fundamental interest, as it will indicate a causal role of GM in AD and provide an excellent model for identifying protective or AD enhancing bacteria. Furthermore, from an animal model perspective, effect size and study power may be increased if mice prior to induction are inoculated with a GM yielding a stronger AD resembling phenotype. Finally, it is of interest to know, whether transfer of a GM from a high responder will increase the Th2 response on the cost of a Th1 response, thereby making the oxazolone model more translational to the initial Th2 dominated response in human AD^28^.

We, therefore, hypothesized that both a high and a low responding oxazolone phenotype can be transferred from conventional mice with the GM to germ-free mice, and that germ-free mice differ in disease sensitivity from conventional mice.

## Material and Methods

### Ethics

The experiments were carried out in accordance with the Danish Act on Animal experimentation (LBK nr 474 of 15/05/2014), which implements the directive 2010/63/EU on the protection of animals used for scientific purposes. The study was approved by the Animal Experimentation Inspectorate, Ministry of Environment and Food, Denmark (license No. 2012-15-2934-00399) and health monitoring was performed according to FELASA guidelines^29^.

### Animals

C57BL/6Ntac mice (n = 16, 7wks) (Taconic, Lille Skensved, Denmark) allowed to acclimate for 1 week were housed in our AAALAC accredited barrier protected facility (Faculty of Health and Medical Sciences, University of Copenhagen, Frederiksberg, Denmark) in open cages without filter lids with free access to an Altromin 1324 diet (Brogaarden, Lynge, Denmark) and tap water.

Nine germ-free C57BL/6Ntac (6 females, 3 males, 7wks) (Taconic, Germantown, NY) were housed in our AAALAC accredited germ-free facility (Faculty of Health and Medical Sciences, University of Copenhagen, Frederiksberg, Denmark) in HEPA-ventilated isolators (PFI systems, Milton Keynes, UK) (pressure 110 pascal, 23 °C) with free access to an irradiated Altromin 1324 diet (Brogaarden) and sterile water. Germ-free status was tested both by culturing and PCR methods^30^. DNA extractions was made with the mobio Powerlyzer Powersoil DNA isolation kit (Mo bio laboratories, inc., Carlsbad, CA) following manufactures instructions.

**Figure 1:**
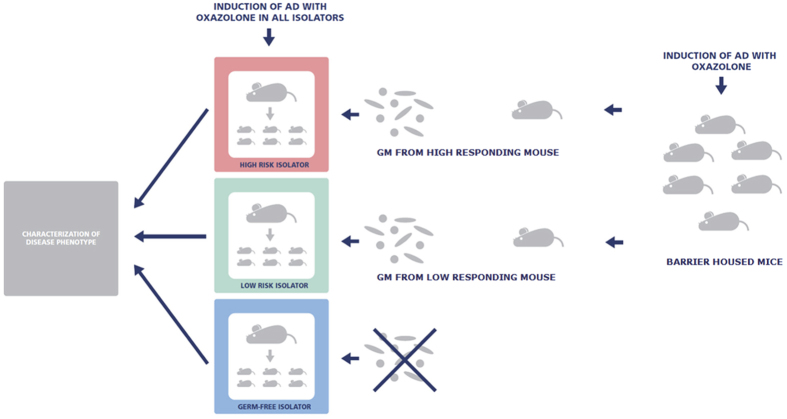
Study setup. Germ-free C57BL/6 dams in three different isolators were inoculated with fecal gut microbiota from the lowest responding conventional mouse (LOW), the highest responding conventional mouse (HIGH) or not inoculated at all. The off-spring of the inoculated dams develops oxazolone induced dermatitis on the ears at the age of eight weeks.

First, dermatitis was induced on the ears of all the barrier housed (CONV) mice. Based upon an overall evaluation of the dermatitis score, ear thickness, and concentrations of IFN-γ, IL-10, IL-1β, IL-2, IL-5, IL-6, and TNF-α, a high and low-responding donor were selected (Table S1). Next, feces from the donors was suspended in phosphate-buffered saline and given by oral gavage to pregnant germ-free dams in two different isolators, and subsequently the off-spring of these (n = 13) (HIGH and LOW mice) as well as germ-free off-spring (n = 12) in a third isolator was used for induction of dermatitis as done for the CONV group (Fig. 1).

### Induction of skin inflammation

All mice (8wks), were sensitized once with 0.8% (w/v) 4-ethoxymethylene-2-phenyl-2-oxazolin-5-one (Oxazolone)(Sigma-Aldrich, St. Louis, MO, USA) dissolved 4:1 in acetone (Emsure, MerckChemicals, Germany) and olive oil (Organic extra virgin olive oil, Svansø, Denmark) with a pipette on both sides of the right ear. After one week the mice were challenged on days 0, 3, 7, 10, 12, 14, 16, 18 and 20 with a fresh solution of 0.4% (w/v) oxazolone dissolved in acetone and olive oil 4:1 on both sides of the right ear. All mice were sacrificed and sampled on day 21. The dosage of oxazolone was based upon an in-house pilot study as well as a previous study showing correlations between the GM and ear tissue concentrations of inflammatory cytokines in the oxazolone model^24^.

### Clinical Scores

At euthanasia the inflamed ear was macroscopically scored blindly by two independent persons for each of the signs 1) flare haemorrhage, 2) oedema, 3) excoriation and erosion, and 4) incrustation and xerosis as follows: 0 = no sign; 1 = mild; 2 = moderate; or 3 = severe^31^ adding the sum of the mean between the two scores as the total dermatitis score. Ear thickness was recorded on day 21 by using a micrometer (Mitutoyo Low Force Caliper Series 573, Aurora, Illinois); each measurement was repeated twice and the calculated mean used. The same person performed all measurements to ensure similar pressure and placement of the micrometer.

### Serum IgE

Orbital blood was collected terminally under anesthesia with 25% Hypnorm (Fentanyl/Fluanison, Vetapharma, Leeds, UK) and 25% Midazolam Hameln (5 mg/ml, Matrix Pharmaceuticals, Hellerup, Denmark) 0.1 ml pr. 10 g BW. Serum IgE concentration was determined in a 1:20 dilution with the mouse IgE ELISA kit from Bethyl Laboratories (Montgomery, TX), following manufacturer’s instructions.

### Ear tissue cytokines

An 8 mm biopsy punch (Kruuse, Langeskov, Denmark) from the inflamed ear was frozen in liquid nitrogen and stored at −80 degrees. The day of analysis, the ear tissue was thawed on ice and homogenized in 300 μl lysis buffer (stock solution contained 10 ml Tris lysis buffer (Mesoscale Discovery, Rockville, MD), 100 μl phosphatase inhibitor 1, 100 μl phosphatase inhibitor 2 and 200 μl protease inhibitor (MSD inhibitor pack, Mesoscale Discovery)) using a tissue blender (Polytron® PT 1200 E, Kinematica AG, Lucerne, Switzerland). The homogenized tissue was centrifuged (7500 g; 5 min) and the supernatant used for analyzing the amount of IFNγ, IL1β, IL2, IL4, IL5, IL6, keratinocyte-derived chemokine/growth-related oncogene (KC/GRO), IL10, IL12p70, and TNFα (Proinflammatory Panel 1 (mouse) kits, Vplex (Mesocale Discovery)) and IL-13 (mouse IL-13 assay ultra-sensitive kit (Mesoscale Discovery)) according to manufactures instructions. Results were read on a MESO QuickPlex SQ 120 (Mesoscale Discovery).

### High throughput sequencing of the gut microbiota

The fecal microbiota of all groups were determined using tag-encoded 16S rRNA gene (V3-V4-region) MiSeq-based (2 × 250 PE) (Illumina, San Diego, CA) high throughput sequencing. DNA extraction was made with mobio Powerlyzer Powersoil DNA isolation kit (Mo bio Laboratories). DNA storage conditions, sequencing library preparation and sequencing steps were conducted as previously described^30^.

The raw dataset containing pair-ended reads with corresponding quality scores were merged and trimmed using fastq_mergepairs and fastq_filter scripts implemented in the UPARSE pipeline setting minimum overlap length to 10 bp. The minimum length of merged reads was 250 bp. The max expected error E = 2.0, and first truncating position with quality score N ≤ 4. Dataset from chimeric reads purging and constructing *de novo* Operational Taxonomic Units (OTU) were conducted UPARSE pipeline^32^. The green genes (13.8) 16S rRNA gene collection was used as a reference database. Quantitative Insight Into Microbial Ecology (QIIME) open source software package (1.7.0, 1.8.0 and 1.9.1)^33^,^34^ was used for analysis.

Alpha and beta diversity analysis was performed as previously described using iterative subsampling (36,000 reads/sample)^35^. Permanova (compare_categories.py, Qiime 1.8.0) and PermanovaG (the Generalized UniFrac R package; GUniFrac)^36^ were used to evaluate group differences using weighted, unweighted and generalized uniFrac^37^ distance matrices respectively. All distance matrices were generated based on rarefied (36,000 reads/sample) OTU tables. The relative distribution of the genera registered was calculated for unified and summarized in the genus level OTU tables.

The G test of independence (q_test) and ANOVA determined respectively: qualitative (presence/absence) and quantitative (relative abundance) association of OTUs with given category. Parametric student’s t-test incorporated in group_significance script (QIIME v9.1) was used to test frequencies of species level OTUs between the “high” and the “low” categories. The probability (p-value) was calculated based on random subsampled OTU tables rarefied to 36,000 per sample.

### GM correlation analysis

Correlations between disease parameters and genera relative abundance were verified with the Pearson’s product-moment correlation coefficient (QIIME 1.7.0), based on 1000 rarefied OTU tables unified to an equal number of reads per sample (36,000). Correlations still significant after randomly removing three subsequent observations were considered valid.

### Statistics

GraphPad Prism version 6.03 (GraphPad Software, San Diego, CA) was used for statistical analysis, and p values less than 0.05 were considered significant. Differences were estimated by one-way ANOVA test with post hoc t-tests for multiple testing or by Kruskal-Wallis test with post hoc Mann-Whitney U-test for multiple testing for data that either did not follow Gaussian distributions or did not have equal variances in the groups. Brown-Forsythe’s test was used to compare differences in variances and ranges. Regressions on the correlation between different parameters, the GM excluded, were made as fitted line plots in Minitab 17 (Minitab Ltd., Coventry, UK).

## Results

In this study, we tried to transfer sensitivity to oxazolone induced dermatitis with the GM from a high and low responding donor to two groups of germfree mice. A third group of mice was kept germfree to investigate the role of bacteria in oxazolone induced dermatitis.

### Immunological parameters

**Figure 2:**
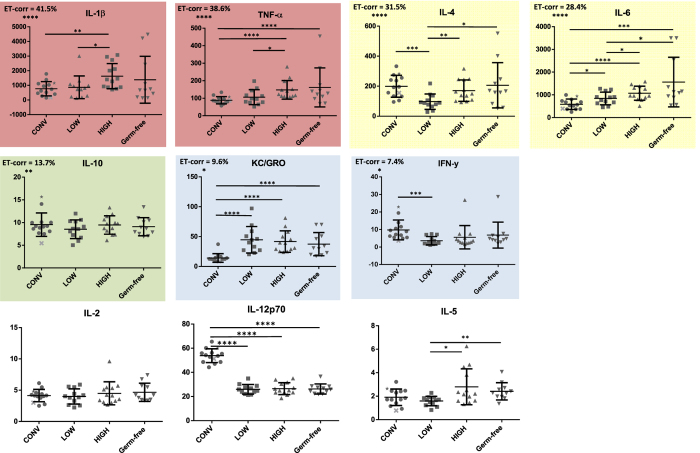
Ear tissue cytokine concentrations. Graphs illustrate cytokine responses (pg/ml) with SD in ear tissue from mice with oxazolone induced dermatitis. CONV: Mice randomly selected from a conventionally barrier-protected colony. CONV mouse marked with ★ = Donor HIGH. CONV mouse marked with **X** = Donor LOW. LOW: Ex-germ-free mice inoculated with the gut microbiota from the lowest responding CONV mouse; HIGH: Ex-germ-free mice inoculated with the gut microbiota from the highest responding CONV mouse; Germfree: Germ-free mice. Highlights reflects the correlation to the clinical score ‘Ear Thickness’ (ET) with the adjusted correlation coefficient (cc) stated with the significance level, i.e. red highlight: cc > 35%; yellow highlight: cc > 25%; green highlight; cc ≥ 15%; blue highlight: cc < 15%; no highlight: not significant. Significance lines indicate differences between means of all groups compared by ANOVA with subsequent t-tests for data with equal variances and by Kruskall-Wallis and Mann-Whitney test for data not showing equal variances. Data from IL-13 is not shown due to concentrations under detection level of the assay. *p < 0.05; **p < 0.01; ***p < 0.001; ****p < 0.0001.

We found that the most relevant cytokines for the oxazolone model were IL-1β, TNFα, IL-4, and IL-6, which shared more than 25% of their variation with the variation in ear thickness in all dermatitis induced mice (Fig. 2). For all of these cytokines, as well as IL-5, the HIGH mice had a higher level in ear tissue than the LOW mice. IL-1β, TNFα, IL-6 and KC/GRO were also higher in HIGH mice compared to the CONV mice, while all germfree derived mice (HIGH, LOW and germfree) had lower concentrations of IL-12p70 compared to the CONV mice (Fig. 2). Data from IL-13 are not shown due to concentrations under detection level of the assay.

Generally, the CONV mice had the lowest variation (TNF-α, KC/GRO) and the germ-free mice had the highest variation (IL-1β, IL-4, IL-6). The inter-individual variation was not lowered in the HIGH and LOW mice inoculated with a uniform GM (Table S2).

### Clinical parameters and serum IgE concentration

**Figure 3:**
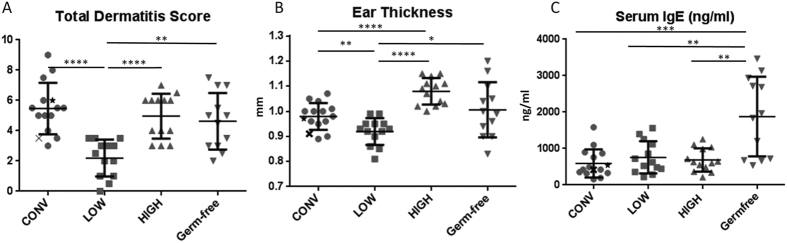
Dermatitis score, Ear thickness and Serum IgE concentration. Graphs illustrate median and range for Total dermatitis score (**A**) and mean and standard deviation for ear thickness (**B**; mm) from ears as well as serum IgE (C; ng/ml) of mice with oxazolone induced dermatitis. CONV: Mice randomly selected from a conventionally barrier-protected colony. CONV mouse marked with ★ = Donor HIGH. CONV mouse marked with **X** = Donor LOW; LOW: Ex-germ-free mice inoculated with the gut microbiota from the lowest responding CONV mouse; HIGH: Ex-germ-free mice inoculated with the gut microbiota from the highest responding CONV mouse; Germfree: Germ-free mice. Significant lines indicate differences between means of all groups compared by ANOVA with subsequent t-tests for data with equal variances and by Kruskall-Wallis and Mann-Whitney test for data not showing equal variances. Differences in dermatitis score (**A**) is calculated by Kruskall-Wallis and Mann-Whitney test. *p < 0.05; **p < 0.01; ***p < 0.001; ****p < 0.0001.

The LOW mice had lower dermatitis score compared to all other groups which did not differ in dermatitis score (Fig. 3A). There were no significant differences in the size of the ranges between the groups (Table S2). The HIGH mice had thicker and the LOW mice had thinner ears compared to both CONV and germ-free mice (Fig. 3B). The germ-free mice expressed a higher variation in ear thickness compared to the other groups (Table S2).

Germ-free mice had significantly higher levels and ranges of serum IgE compared to the other groups, in which no differences were evident (Fig. 3C and Table S2).

### Gut microbiota upon transfer to germ-free mice

**Figure 4:**
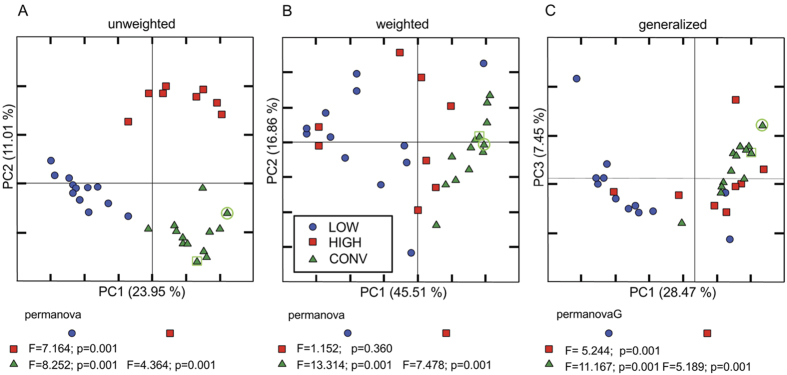
PCoA plots of gut microbiota clustering. Projection of differences in the gut microbiota composition between mice with oxazolone induced dermatitis. CONV: Mice randomly selected from a conventionally barrier-protected colony; LOW: Ex-germ-free mice inoculated with the gut microbiota from the lowest responding SPF mouse (marked with a green circle); HIGH: Ex-germ-free mice inoculated with the gut microbiota from the highest responding CONV mouse (marked with a green square); Germfree: Germ-free mice. PCoA plots based on unweighted (**A**), weighted (**B**) and generalised UniFrac distance matrices calculated from 10 rarefied (36,000 reads/sample) OTU tables. Group differences between the three categories were based on PermanovaG.

**Figure 5:**
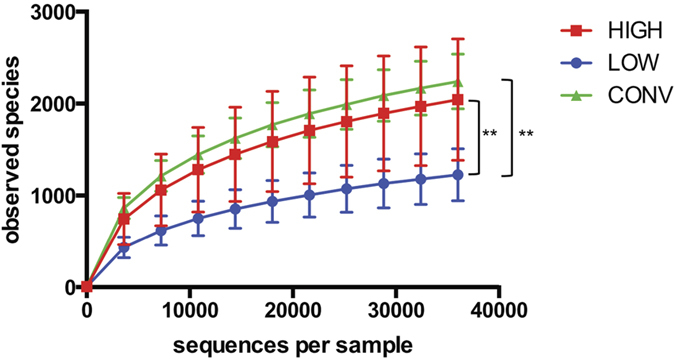
Diversity indexes. Rarefaction curves based on observed species index (97% sequence similarity threshold), Chao1 and Shannons diversity index. Alpha diversity measure expressed with observed species and chao1 indexes revealed significantly reduced (p < 0.01) diversity in ex-germ-free mice inoculated with the gut microbiota from the lowest responding CONV mouse (LOW) compared to ex-germ-free mice inoculated with the gut microbiota from the highest responding CONV mouse (HIGH) as well as to barrier protected mice (CONV). Shannon diversity index only revealed a reduced (p < 0.01) diversity in LOW mice compared to CONV mice. **p < 0.01.

Sequences purged from chimeric reads yielded 3,571,114 giving an average of 101,998 sequences per sample (min = 42,442; max = 223,427; SD = 43,185 with a mean sequence length of 451 bp (±25 bp). Five samples were discarded due to low read number (<300 reads). GM transfer resulted in two new GM profiles clearly different from each other and from the conventional mice (Fig. 4A,B and C). The CONV and HIGH mice had comparable alpha diversity indexes, while the LOW mice had significantly lower diversity compared to CONV and HIGH according to observed species and chao1 indexes (p < 0.01), while the LOW Shannon diversity index was only significantly lower compared to CONV (p < 0.01)(Fig. 5). This difference was mostly due to the LOW mice missing a number of Clostridiales assigned OTUs. The HIGH mice did not harbor the specific murine bacterium *Mucispirillum schaedleri* (Table 1).

The community level differences in relative abundance between CONV, HIGH and LOW groups (Fig. 4B) were also reflected in the relative abundance of several OTUs being significantly different between the groups. Noticeable differences are that the HIGH and LOW mice had a lower abundance of unclassified species belonging to the families of Bacteroidales S24-7 and Lachnospiraceae and higher abundance of *Lactobacillus* spp. compared to the CONV mice (Table 2A). When comparing the GM of LOW and HIGH mice alone, the GM of HIGH mice was to a higher degree dominated by species belonging to the family of Lachnospiraceae, as well as *Bacteroides uniformis* and an unclassified genus of Rikenellaceae (Table 2B). In contrast, the LOW mice tended to have more unclassified *Bacteroides* spp. compared to the HIGH mice (p = 0.008, FDR = 0.059).

These differences evident between the inoculated groups were not present in the donor mice, in which there was no difference in the occurrence or abundance of Lachnospiraceae spp., *Bacteroides* spp., Rikenellaceae spp. or *M. schaedleri* (Table S3). The LOW donor seemed to have a lower abundance of *Lactobacillus* compared to the HIGH donor. This was opposite in the colonized groups in which the LOW mice tended to have more *Lactobacillus* than the HIGH mice. However, a pronounced abundance of *Lactobacillus* is evident in both the colonized groups (Table 2A).

The relative abundance of several species correlated with various clinical and immunological parameters. Even after random removal of a data point three times, significant correlations, mostly within the phylum Firmicutes, were found (Figure S1). Most strikingly, ear thickness in CONV mice showed a strong correlation to *Mollicutes* spp., *Enterococcus* spp., and *Mucispirillum* spp.

## Discussion

The HIGH and the LOW phenotypes from the conventional mice were clearly transferred to germ-free mice, as there was a higher expression of dermatitis score, ear thickness, IL-1β, TNFα, IL-4, and IL-6 in the HIGH mice compared to the LOW mice, and for most of these also higher than the CONV mice. GM transfer in general had very little impact on inter-individual variation within the groups in this model. However, the germ-free mice had the highest variation in ear thickness, IgE, IL-1β, TNFα, IL-4, and IL-6, so most likely GM secures a uniform response in these parameters. In study designs, group sizes may be reduced by using tailor-made mice inoculated with a high responder GM due to the increased clinical effect size.

The strong correlation between macrophage produced cytokines IL-1 β^38^, TNFα^39^, and IL-6^40^ and ear thickness indicate a GM impact on macrophage activity with subsequent impact on inflammation and T-cell proliferation. The IL-1β augmented TNFα signaling due to up-regulation of TNFα secretion and surface receptor expression in macrophages is triggered by Gram negative bacterial wall lipopolysaccharides (LPS)^41^, and is an important part of antimicrobial immunity^42^. IL-4 from Th2 cells^43^ induce IgE isotype switch in B-cells^44^, which is closely linked to atopic allergy diseases^45^,^46^. GM transfer therefore seems to increase T cell responses in the HIGH mice, and their increased level of the Th2 cytokine IL-5^43^ indicates a higher Th2 response. Moreover, IL-1β, highly correlating with ear thickness, promotes the formation of IL-17 producing Th17 cells^47^; a key element in the Th2 activation in AD in mice^48^. The low level of IL12p70 in GM transplanted mice indicates that the role of Th1 cells was decreased by GM transfer. IgE responses did not seem to differ between neither the mice with different GM, nor in the donor mice. Interestingly, germ-free mice had a much higher and more varying IgE production compared to all colonized mice strongly supporting the protective role of bacteria in type 1 hypersensitivity reactions known from other murine allergy models^49^,^50^.

Neither the GM of HIGH, nor LOW mice clustered with their donors. GM transfer dramatically increased the relative abundance of Firmicutes; primarily due to an increase in *Lactobacillus* spp. Previously, we found that when germ-free mice were inoculated at weaning they clustered with the inoculum later in life, but we also showed that timing of inoculation is important for transferring GM profiles^51^. It is, however, likely that there will be changes in the GM, when the mothers are inoculated, when the offspring are born, and over time from birth to termination that could explain why clustering is not observed between inoculum and recipients. Further, it cannot be ruled out that short exposure to oxygen during transfer, influences the inoculum giving oxygen-tolerant species such as *Lactobacillus* spp. a competitive advantage when the GM of the inoculated mice is established.

Remarkably, germ-free mice responded highly in key parameters, such as total dermatitis score, ear thickness, TNFα, IL-4, and IL-5. As the LOW mice had the lowest GM diversity compared to HIGH and CONV mice, the high response of the germ-free mice indicates a GM impact on AD in mice to be a question of the presence of protective bacteria rather than a question of diversity. Bacteria present in the LOW mice in contrast to the HIGH mice (Table 1) were *M. schaedleri, Blautia producta* and an unclassified species of Erysipelotrichaceae. None of these are known for specific protecting roles against AD. *M. schaedleri* of the Deferribacteres phylum is not found in the human gut, but it may in mice have abundance up to 1%, and it has been used for decades as a member of the altered Schaedler flora (ASF 457)^52^,^53^,^54^. However, in the CONV mice there was a strong positive correlation between the abundance of *M. schaedleri* and ear thickness, so it is not the most likely AD-protecting candidate.

The two colonized groups differed in the relative abundance of a range of bacteria. For example, Lachnospiraceae spp. was more abundant in the HIGH mice, which may increase AD response, as children suffering from AD tend to have a higher abundance of Clostridia^14^,^21^,^55^. On the other hand, LOW mice showed a tendency to have a higher abundance of *Lactobacillus* spp., which was only significant if a general outlier in the LOW group was removed (p = 0.039 with outlier removed), and *Lactobacillus* spp. may have a possible protective effect against AD, as previous studies have shown that different species of dietary *Lactobacillus* may relieve AD symptoms in murine models^56^,^57^,^58^,^59^,^60^.

LOW mice compared to HIGH mice showed a tendency (p = 0.008, FDR-corrected p = 0.059) for higher abundance of the genus *Bacteroides*, which may have a preventive effect, as a previous study has shown that *Bacteroides fragilis* is anti-inflammatory and contributes to development of host immunity^61^. Further, monocolonization with *B. fragillis* lowered the amount of IL-4 production from CD4^+^ T cells in germ-free mice^62^. Therefore, the lower concentration of IL-4 in the LOW mice could be mediated by the higher abundance of *Bacteroides*. Compared to the LOW mice, HIGH mice showed a greater abundance of another *Bacteroides* fraction identified as *Bacteroides uniformis*. Administration of *B. uniformis* has been shown to ameliorate obesity and the associated immune alteration^63^. Obesity is Th1 dominated^64^, in contrast to the Th2 dominated AD^3^,^4^,^5^. Consequently, the higher abundance of *B. uniformis* in the HIGH mice may alter the Th1/Th2 balance, thereby contributing to Th2 predominance in the HIGH mice.

This study shows that the expression of oxazolone induced dermatitis in mice can be influenced solely by GM interventions. As mentioned in the introduction, patients with AD have compositional changes in their GM^13^,^14^,^15^,^16^,^21^, which indicates that GM intervention as treatment or prevention strategy could have an alleviating effect on AD. GM intervention would be a good alternative to the present treatment of AD, which now comprises mainly of topical corticosteroids, which can cause adverse skin side effects^65^.

The diet has been shown to be an important driver of GM composition and function^66^,^67^,^68^, so a dietary intervention could be a possible and easy accessible approach to change the GM in patients with AD, resembling that of healthy individuals. A more complicated, yet possible, method is fecal transplantation from healthy individuals to patients with AD. Fecal transplantation has recently shown to be effective against other GM associated diseases e.g. Inflammatory Bowel Disease^69^,^70^,^71^ and *Clostridium difficile* infection^72^, so it is not unlikely that it could influence the expression of AD. The effect of fecal transplantation may decrease over time in a certain fraction of the patients, e.g. as observed for ulcerative colitis^73^, but since AD is considered a childhood disease, where symptoms often diminish in adulthood, this will most likely be a minor problem. Before fecal transplantation can be an option for treatment of AD, it would require much more research to identify biomarkers and protective bacteria to find the optimal donors.

In conclusion, a high and a low responding phenotype of AD can be transferred with the GM to germ-free mice. The use of a high responding donor improves the applicability of the inoculated mice as models, because they develop increased ear thickness, higher IL-1β, and TNFα concentrations in ear tissue, as well as a more Th2 dominated response.

## Additional Information

**How to cite this article:** Zachariassen, L. F. *et al*. Sensitivity to oxazolone induced dermatitis is transferable with gut microbiota in mice. *Sci. Rep.* **7**, 44385; doi: 10.1038/srep44385 (2017).

**Publisher's note:** Springer Nature remains neutral with regard to jurisdictional claims in published maps and institutional affiliations.

## Supplementary Material

Supplementary Tables and Figure S1

## Figures and Tables

**Table 1:** G test showing the qualitative (presence/absence) association of Operational Taxonomic Units (OTU).

phylum	class	order	family	genus	species	significantly different after FDR correction	Number of species level OTUs with significantly skewed distribution in:					
							CONV		LOW		HIGH	
							more than expected	less than expected	more than expected	less than expected	more than expected	less than expected
Bacteroidetes	Bacteroidia	Bacteroidales	S24-7	Unclassified	Unclassified	102	90	12	17	85	36	66
Deferribacteres	Deferribacteres	Deferribacterales	Deferribacteraceae	*Mucispirillum*	*schaedleri*	1	1	0	1	0	0	1
Firmicutes	Bacilli	Lactobacillales	Lactobacillaceae	*Lactobacillus*	Unclassified	61	0	61	61	0	59	2
Firmicutes	Clostridia	Clostridiales	Lachnospiraceae	*Blautia*	*producta*	1	0	1	1	0	0	1
Firmicutes	Clostridia	Clostridiales	Lachnospiraceae	*Blautia*	Unclassified	2	2	0	0	2	2	0
Firmicutes	Clostridia	Clostridiales	Lachnospiraceae	*Coprococcus*	Unclassified	2	2	0	0	2	1	1
Firmicutes	Clostridia	Clostridiales	Lachnospiraceae	*Lachnobacterium*	Unclassified	1	1	0	0	1	0	1
Firmicutes	Clostridia	Clostridiales	Lachnospiraceae	*Ruminococcus*	*gnavus*	12	10	2	0	12	12	0
Firmicutes	Clostridia	Clostridiales	Lachnospiraceae	Unclassified	Unclassified	145	100	45	13	132	114	31
Firmicutes	Clostridia	Clostridiales	Peptococcaceae	*rc4*-*4*	Unclassified	1	1	0	0	1	0	1
Firmicutes	Clostridia	Clostridiales	Ruminococcaceae	*Ruminococcus*	Unclassified	2	2	0	0	2	0	2
Firmicutes	Clostridia	Clostridiales	Ruminococcaceae	Unclassified	Unclassified	5	5	0	0	5	1	4
Firmicutes	Clostridia	Clostridiales	Unclassified	Unclassified	Unclassified	296	223	73	87	209	166	130
Firmicutes	Erysipelotrichi	Erysipelotrichales	Erysipelotrichaceae	Unclassified	Unclassified	1	1	0	1	0	0	1
Tenericutes	Mollicutes	Anaeroplasmatales	Anaeroplasmataceae	*Anaeroplasma*	Unclassified	1	1	0	0	1	0	1

Compilation of the g test results based on the subsampled (36,000 reads/sample) species level OTUs in conventional (CONV), as well as ex-germ-free mice inoculated with gut microbiota from either a high responder (HIGH) or a low responder (LOW) in relation to atopic dermatitis. In total 633 OTUs were significantly associated with one of three categories. All those OTUs could be categorized into 15 taxonomic groups presented in the table. For example (firs row): In total 102 OTUs, all representing S24-7 family, presented significantly skewed distribution between the three categories. 90 of these OTUs were overrepresented (more than expected) within the SPF category, while 12 remaining OTUs were underrepresented in this group etc. The False Discovery Rate (FDR) represents the probability after correction with FDR where the raw p-values are firstly ranked from low to high and then each p-value is multiplied by the number of tests divided by this rank. Taxa denoted as “unclassified” means that the reference database does not have an official taxonomy for this cluster. Taxa denoted as “Other” indicates ambiguity in the assignment meaning that more than one taxon could be assigned to this cluster at given taxonomic level.

**Table 2:** Differences in relative abundance of given OTU (97% sequence similarity).

phylum	class	order	family	genus	species	p value	FDR	HIGH %	LOW %	CONV %
**(A**)										
Actinobacteria	Coriobacteriia	Coriobacteriales	Coriobacteriaceae	*Adlercreutzia*	Unclassified	0.00109	0.00638	0.01	0.01	0.03
Bacteroidetes	Bacteroidia	Bacteroidales	Bacteroidaceae	*Bacteroides*	*uniformis*	0.00398	0.01266	0.08	0.02	0.06
Bacteroidetes	Bacteroidia	Bacteroidales	Rikenellaceae	Unclassified	Unclassified	0.01223	0.02853	0.03	0.01	0.05
Bacteroidetes	Bacteroidia	Bacteroidales	S24-7	Unclassified	Unclassified	0.01288	0.02817	15.50	11.11	25.86
Firmicutes	Bacilli	Lactobacillales	Lactobacillaceae	*Lactobacillus*	Other	0.00116	0.00580	0.70	0.38	1.87
Firmicutes	Bacilli	Lactobacillales	Lactobacillaceae	*Lactobacillus*	*reuteri*	0.00684	0.01710	1.27	0.65	2.62
Firmicutes	Bacilli	Lactobacillales	Lactobacillaceae	*Lactobacillus*	Unclassified	0.00009	0.00104	43.09	64.21	12.93
Firmicutes	Clostridia	Clostridiales	Clostridiaceae	*Unclassified*	Unclassified	0.01893	0.03313	0.01	0.01	0.02
Firmicutes	Clostridia	Clostridiales	Dehalobacteriaceae	*Dehalobacterium*	Unclassified	0.02601	0.04335	0.01	0.00	0.02
Firmicutes	Clostridia	Clostridiales	Lachnospiraceae	[*Ruminococcus*]	*gnavus*	0.00306	0.01071	1.89	0.29	1.22
Firmicutes	Clostridia	Clostridiales	Lachnospiraceae	*Blautia*	Unclassified	0.00019	0.00162	0.05	0.00	0.01
Firmicutes	Clostridia	Clostridiales	Lachnospiraceae	*Coprococcus*	Unclassified	0.00056	0.00390	0.14	0.04	0.25
Firmicutes	Clostridia	Clostridiales	Lachnospiraceae	*Dorea*	Unclassified	0.01843	0.03395	0.00	0.00	0.00
Firmicutes	Clostridia	Clostridiales	Lachnospiraceae	Other	Other	0.00236	0.00919	0.41	0.09	0.89
Firmicutes	Clostridia	Clostridiales	Lachnospiraceae	*Roseburia*	Unclassified	0.01474	0.02867	0.01	0.00	0.00
Firmicutes	Clostridia	Clostridiales	Lachnospiraceae	Unclassified	Unclassified	0.00141	0.00615	13.25	2.76	13.77
Firmicutes	Clostridia	Clostridiales	Other	Other	Other	0.00415	0.01211	0.79	0.24	1.69
Firmicutes	Clostridia	Clostridiales	Peptococcaceae	*rc4*-*4*	Unclassified	0.00003	0.00052	0.09	0.05	0.20
Firmicutes	Clostridia	Clostridiales	Ruminococcaceae	Unclassified	Unclassified	0.00003	0.00097	0.48	0.25	1.00
Tenericutes	Mollicutes	RF39	Unclassified	Unclassified	Unclassified	0.00427	0.01149	0.00	0.00	0.02
**phylum**	**class**	**order**	**family**	**genus**	**species**	**p value**	**FDR**	**HIGH %**	**LOW %**	
**(B**)										
Bacteroidetes	Bacteroidia	Bacteroidales	Bacteroidaceae	*Bacteroides*	*uniformis*	0.00110	0.02919	0.07	0.02	
Bacteroidetes	Bacteroidia	Bacteroidales	Rikenellaceae	Unclassified	Unclassified	0.00204	0.03001	0.03	0.01	
Firmicutes	Clostridia	Clostridiales	Lachnospiraceae	[*Ruminococcus*]	*gnavus*	0.00325	0.03227	1.89	0.30	
Firmicutes	Clostridia	Clostridiales	Lachnospiraceae	*Blautia*	*producta*	0.00365	0.03227	0.00	0.01	
Firmicutes	Clostridia	Clostridiales	Lachnospiraceae	*Blautia*	Unclassified	0.00097	0.02919	0.05	0.00	
Firmicutes	Clostridia	Clostridiales	Lachnospiraceae	Unclassified	Unclassified	0.00227	0.03001	13.27	2.78	
Bacteroidetes	Bacteroidia	Bacteroidales	Bacteroidaceae	*Bacteroides*	Unclassified	0.00782	0.05918	0.43	1.15	
Firmicutes	Clostridia	Clostridiales	Lachnospiraceae	Unclassified	Unclassified	0.01148	0.07384	0.41	0.09	
Firmicutes	Clostridia	Clostridiales	Lachnospiraceae	*Roseburia*	Unclassified	0.01254	0.07384	0.01	0.00	
Firmicutes	Clostridia	Clostridiales	Unclassified	Unclassified	Unclassified	0.03022	0.16014	0.80	0.25	
Firmicutes	Clostridia	Clostridiales	Lachnospiraceae	*Coprococcus*	Unclassified	0.03533	0.17024	0.15	0.04	
Firmicutes	Clostridia	Clostridiales	Dehalobacteriaceae	*Dehalobacterium*	Unclassified	0.04211	0.186	0.01	0.00	

CONV: Mice randomly selected from a conventionally barrier-protected colony. LOW: Ex-germ-free mice inoculated with the gut microbiota from the lowest responding CONV mouse; HIGH: Ex-germ-free mice inoculated with the gut microbiota from the highest responding CONV mouse analyzed with A) ANOVA and B) HIGH and LOW analyzed with parametric t-test.

The p-value was calculated based on 1000 subsampled OTU tables rarefied to 36,000 per sample. The False Discovery Rate (FDR) represents the probability after correction with FDR where the raw p-values are firstly ranked from low to high and then each p-value is multiplied by the number of tests divided by this rank. “Unclassified” stands for taxa having no official taxonomy in the database. Taxa denoted as “Other” indicates ambiguity in the assignment, meaning that more than one taxon could be assigned to this cluster at given taxonomic level. Taxa mentioned in the square brackets indicate a proposed taxonomy.
