# Supplementary material for: Sensitivity to oxazolone induced dermatitis is transferable with gut microbiota in mice
Source: Sci Rep. 2017 Mar 14;7:44385. doi: 10.1038/srep44385 (PMC5349591; doi:10.1038/srep44385)

Supplementary information for manuscript:

Sensitivity to oxazolone induced dermatitis is transferable with gut microbiota in mice

Line Fisker Zachariassen[*#], Lukasz Krych[†], Kåre Engkilde[‡], Dennis Sandris Nielsen[†], Witold Kot[§], Camilla Hartmann Friis Hansen[#], Axel Kornerup Hansen[#].

Table S1. Parameters applied for selection of mouse No 44 as HIGH responder and Mouse No 47 as LOW responder in a study of oxazolone induced dermatitis. Red: Values > 75 % quartile; Green: Values < 25 % quartile.

Animal number	Sex	Dermatitis score	Ear thickness	IgE	IFN-y	IL-10	IL-12p70	IL-1b	IL-2	IL-4	IL-5	IL-6	KC/GRO	TNF-a
			mm	ng/ml	pg/ml									
39	♂	3.5	1.04	191	13.81	14.09	48.56	464.10	4.38	241.37	3.22	995.52	12.31	72.56
40	♀	4	0.96	159	19.31	10.09	65.41	1389.71	4.57	196.23	2.41	681.34	16.36	95.15
41	♂	5	0.89	352	11.48	9.97	53.62	732.52	5.47	236.73	2.44	799.95	12.61	87.07
42	♀	5.5	1	900	6.65	8.08	55.35	1030.70	4.25	274.65	1.70	677.38	8.87	84.31
43	♂	3	0.95	317	6.12	9.09	56.15	269.11	4.15	156.73	2.47	518.11	14.02	56.77
44	♀	6	0.97	541	22.75	15.66	60.00	1153.90	4.80	283.02	2.64	941.38	10.49	107.37
45	♂	5	0.96	1585	4.76	9.22	44.24	1115.59	6.12	332.91	2.30	731.78	11.87	78.85
46	♀	5.5	1	491	8.79	10.01	48.55	523.11	2.74	95.42	1.41	368.92	12.45	67.22
47	♂	3.5	0.91	419	3.45	5.45	51.21	170.09	3.00	155.39	0.78	390.58	8.67	85.90
48	♀	9	1.07	1099	10.42	8.85	54.25	946.22	3.77	255.99	1.84	564.46	14.63	118.24
49	♂	5	0.9	422	5.33	7.51	60.17	149.08	4.00	104.61	0.92	246.56	12.26	80.08
50	♀	7.5	1.05	328	7.01	7.67	47.06	622.50	4.03	131.92	1.72	368.06	8.53	100.86
51	♀	5.5	0.98	758	-	-	-	-	-	-	-	-	-	-
52	♂	6	1	423	5.31	9.24	52.86	402.89	2.51	133.64	1.16	371.4934	18.47	72.92
53	♀	8	1.01	868	9.35	8.63	55.86	1784.44	4.13	192.94	1.72	535.82	37.21	133.98

Table S2. Differences between variances of groups where CONV: Mice randomly selected from a conventionally barrier-protected colony; LOW: Ex-germ-free mice inoculated with the gut microbiota from the lowest responding SPF mouse; HIGH: Ex-germ-free mice inoculated with the gut microbiota from the highest responding CONV mouse; Germfree: Germ-free mice. For comparison of variances F-tests were used. Dermatitis score, IL-2, IL-10 and IL-12p70 is not shown in the table due to equal variances.
NS = non significant.
* p < 0.05; ** p < 0.01; *** p < 0.001; **** p < 0.0001

Ear Thickness		CONV	LOW	HIGH	GERMFREE
	CONV	-	NS	NS	*
	LOW		-	NS	*
	HIGH			-	*
	GERMFREE				-

IgE		CONV	LOW	HIGH	GERMFREE
	CONV	-	NS	NS	***
	LOW		-	NS	***
	HIGH			-	***
	GERMFREE				-

IL-1b		CONV	LOW	HIGH	GERMFREE
	CONV	-	NS	NS	***
	LOW		-	NS	NS
	HIGH			-	*
	GERMFREE				-

IL-4		CONV	LOW	HIGH	GERMFREE
	CONV	-	NS	NS	*
	LOW		-	NS	**
	HIGH			-	*
	GERMFREE				-

IL-5		CONV	LOW	HIGH	GERMFREE
	CONV	-	NS	**	NS
	LOW		-	****	NS
	HIGH			-	*
	GERMFREE				-

IL-6		CONV	LOW	HIGH	GERMFREE
	CONV	-	NS	NS	****
	LOW		-	NS	****
	HIGH			-	***
	GERMFREE				-

TNF-a		CONV	LOW	HIGH	GERMFREE
	CONV	-	*	**	****
	LOW		-	NS	NS
	HIGH			-	*
	GERMFREE				-

KC/GRO		CONV	LOW	HIGH	GERMFREE
	CONV	-	***	**	**
	LOW		-	NS	NS
	HIGH			-	NS
	GERMFREE				-

IFN-y		CONV	LOW	HIGH	GERMFREE
	CONV	-	**	NS	NS
	LOW		-	**	***
	HIGH			-	NS
	GERMFREE				-

Table S3: Distribution of OTU (97% sequence similarity) in the gut microbiota of the high and low responding donor mice. “Unclassified” stands for taxa having no official taxonomy in the database. Taxa denoted as “Other” indicates ambiguity in the assignment, meaning that more than one taxon could be assigned to this cluster at given taxonomic level. Taxa mentioned in the square brackets indicate a proposed taxonomy.

phylum	class	order	family	genus	species	donor (HIGH)	donor (LOW)
Unassigned	Other	Other	Other	Other	Other	30	55
Actinobacteria	Coriobacteriia	Coriobacteriales	Coriobacteriaceae	*Adlercreutzia*	Unclassified	22	17
Bacteroidetes	Bacteroidia	Bacteroidales	Bacteroidaceae	*Bacteroides*	Unclassified	209	279
Bacteroidetes	Bacteroidia	Bacteroidales	Bacteroidaceae	*Bacteroides*	*Uniformis*	15	17
Bacteroidetes	Bacteroidia	Bacteroidales	Porphyrominadacea	*Parabacteroides*	Unclassified	48	40
Bacteroidetes	Bacteroidia	Bacteroidales	Rikenellaceae	Unclassified	Unclassified	18	15
Bacteroidetes	Bacteroidia	Bacteroidales	S24-7	Unclassified	Unclassified	10882	11002
Deferribacteres	Deferribacteres	Deferribacterales	Deferribacteraceae	*Mucispirillum*	*schaedleri*	3	2
Firmicutes	Bacilli	Lactobacillales	Enterococcaceae	*Enterococcus*	Unclassified	0	12
Firmicutes	Bacilli	Lactobacillales	Lactobacillaceae	*Lactobacillus*	Other	443	82
Firmicutes	Bacilli	Lactobacillales	Lactobacillaceae	*Lactobacillus*	Unclassified	4432	1066
Firmicutes	Bacilli	Lactobacillales	Lactobacillaceae	*Lactobacillus*	*reuteri*	709	124
Firmicutes	Bacilli	Turicibacterales	Turicibacteraceae	*Turicibacter*	Unclassified	0	1
Firmicutes	Clostridia	Clostridiales	Other	Other	Other	203	136
Firmicutes	Clostridia	Clostridiales	Unclassified	Unclassified	Unclassified	13051	14074
Firmicutes	Clostridia	Clostridiales	Clostridiaceae	Unclassified	Unclassified	11	3
Firmicutes	Clostridia	Clostridiales	Dehalobacteriaceae	*Dehalobacterium*	Unclassified	8	12
Firmicutes	Clostridia	Clostridiales	Lachnospiraceae	Other	Other	365	539
Firmicutes	Clostridia	Clostridiales	Lachnospiraceae	Unclassified	Unclassified	4132	5633
Firmicutes	Clostridia	Clostridiales	Lachnospiraceae	*Anaerostipes*	Unclassified	1	0
Firmicutes	Clostridia	Clostridiales	Lachnospiraceae	*Blautia*	Unclassified	1	4
Firmicutes	Clostridia	Clostridiales	Lachnospiraceae	*Blautia*	*producta*	5	2
Firmicutes	Clostridia	Clostridiales	Lachnospiraceae	*Butyrivibrio*	Unclassified	1	2
Firmicutes	Clostridia	Clostridiales	Lachnospiraceae	*Coprococcus*	Unclassified	93	106
Firmicutes	Clostridia	Clostridiales	Lachnospiraceae	*Dorea*	Unclassified	3	8
Firmicutes	Clostridia	Clostridiales	Lachnospiraceae	*Lachnobacterium*	Unclassified	21	84
Firmicutes	Clostridia	Clostridiales	Lachnospiraceae	*Roseburia*	Unclassified	4	8
Firmicutes	Clostridia	Clostridiales	Lachnospiraceae	*[Ruminococcus]*	Other	1	7
Firmicutes	Clostridia	Clostridiales	Lachnospiraceae	*[Ruminococcus]*	Unclassified	4	12
Firmicutes	Clostridia	Clostridiales	Lachnospiraceae	*[Ruminococcus]*	*Gnavus*	423	967
Firmicutes	Clostridia	Clostridiales	Peptococcaceae	*rc4-4*	Unclassified	159	51
Firmicutes	Clostridia	Clostridiales	Ruminococcaceae	Other	Other	4	2
Firmicutes	Clostridia	Clostridiales	Ruminococcaceae	Unclassified	Unclassified	303	420
Firmicutes	Clostridia	Clostridiales	Ruminococcaceae	*Oscillospira*	Unclassified	269	789
Firmicutes	Clostridia	Clostridiales	Ruminococcaceae	*Ruminococcus*	Unclassified	109	390
Firmicutes	Clostridia	Clostridiales	[Mogibacteriaceae]	Unclassified	Unclassified	4	13
Firmicutes	Erysipelotrichi	Erysipelotrichales	Erysipelotrichaceae	Unclassified	Unclassified	3	8
Firmicutes	Erysipelotrichi	Erysipelotrichales	Erysipelotrichaceae	*Coprobacillus*	Unclassified	0	1
Proteobacteria	Alphaproteobacteria	Rickettsiales	Mitochondria	Other	Other	5	2
Proteobacteria	Gammaproteobacteria	Enterobacteriales	Enterobacteriaceae	Unclassified	Unclassified	0	7
Tenericutes	Mollicutes	Anaeroplasmatales	Anaeroplasmataceae	*Anaeroplasma*	*Unclassified*	0	3
Tenericutes	Mollicutes	RF39	Unclassified	Unclassified	Unclassified	6	5

Figure S1

Correlation between and specific parameters of oxazolone induced dermatitis and the abundances of specific species in groups of mice randomly selected from a conventionally barrier-protected colony (CONV), ex-germ-free mice inoculated with the gut microbiota from the highest responding CONV mouse (HIGH), and ex-germ-free mice inoculated with the gut microbiota from the lowest responding SPF mouse (LOW). Only correlations robust to be significant in each of three jacknifings (random removal of a data set) have been shown.

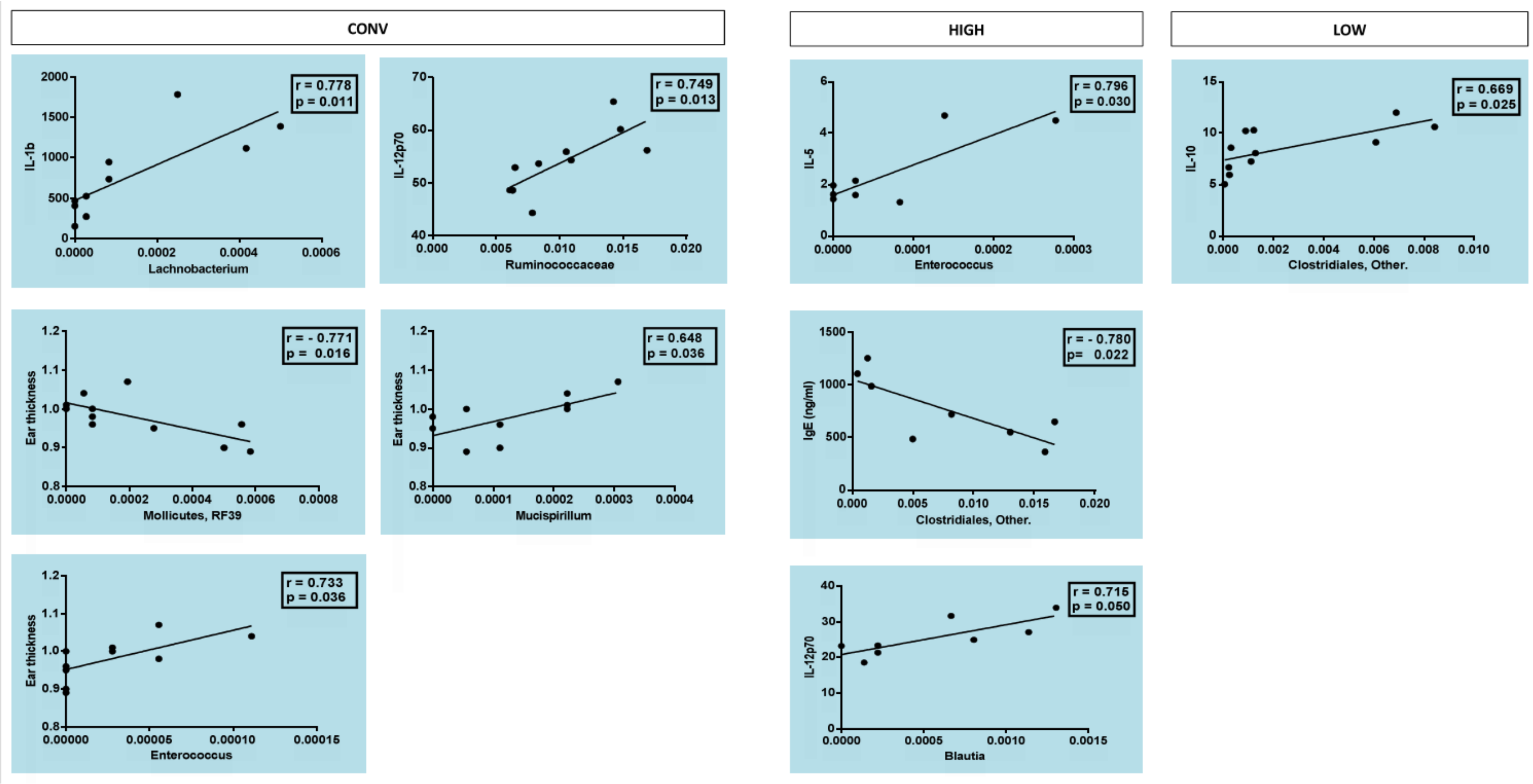

Supplement: Supplementary Tables and Figure S1 [file srep44385-s1.pdf]
